# Skeletal muscle‐specific PGC‐1α‐b overexpression prevents eccentric contraction‐induced muscle injury through an utrophin‐independent pathway in mice

**DOI:** 10.14814/phy2.70743

**Published:** 2026-01-22

**Authors:** Azuma Naito, Nao Tokuda, Nao Yamauchi, Ayaka Niibori, Kazuma Okada, Koichi Himori, Yuki Ashida, Takashi Yamada

**Affiliations:** ^1^ Graduate School of Health Sciences Sapporo Medical University Sapporo Japan; ^2^ The Japan Society for the Promotion of Science (JSPS) Tokyo Japan; ^3^ Institute for Glyco‐Core Research, Tokai National Higher Education and Research System Nagoya Japan; ^4^ Department of Molecular Therapy National Institute of Neuroscience, National Center of Neurology and Psychiatry Tokyo Japan; ^5^ Graduate School of Biomedical and Health Sciences Hiroshima University Hiroshima Japan

**Keywords:** damage resistance, eccentric contraction, fiber type, PGC‐1α‐b, utrophin

## Abstract

Slower oxidative fibers are more resistant to eccentric contraction (ECC)‐induced muscle damage than fast‐twitch glycolytic fibers, but the mechanisms remain unclear. This study investigated the roles of the exercise‐inducible PGC‐1α isoform PGC‐1α‐b and utrophin in protecting against ECC‐induced damage. ECCs were induced by supramaximal electrical stimulation of the left triceps surae in C57BL/6N wild‐type (WT), PGC‐1α‐b transgenic (Tg), utrophin knockout (Utrn KO), and PGC‐1α‐b Tg/Utrn KO mice. Although the proportion of fast‐type myosin heavy chain (MyHC) IIb in the gastrocnemius muscle was modestly lower in PGC‐1α‐b Tg and PGC‐1α‐b Tg/Utrn KO mice than in WT and Utrn KO mice, MyHC IIb remained the predominant isoform. At 3 days post injury (dpi), WT and Utrn KO mice exhibited reduced maximum isometric torque (MIT), Evans blue dye (EBD) staining in MyHC IIb‐positive fibers, and calpain‐1 activation. In contrast, PGC‐1α‐b Tg and PGC‐1α‐b Tg/Utrn KO mice showed substantial MIT recovery at 1 dpi and minimal EBD uptake and calpain‐1 activation at 3 dpi. PGC‐1α‐b Tg muscles also preserved excitation‐contraction coupling proteins and displayed increased mitochondrial markers and integrin α7B expression. Together, our findings suggest that PGC‐1α‐b confers resistance to ECC‐induced muscle damage through a Utrn‐independent mechanism.

## Graphical abstract

PGC‐1α‑b overexpression protects glycolytic muscle fibers from eccentric contraction–induced injury. Eccentric contractions caused marked membrane damage and loss of maximal isometric torque in wild‐type and utrophin‐deficient mice, particularly within MyHC IIb–dominant fibers. In contrast, PGC‑1α‑b transgenic mice, regardless of utrophin expression, showed rapid torque recovery and minimal Evans blue dye uptake. These findings identify PGC‑1α‑b as a potent, utrophin‐independent protector against ECC‑induced muscle damage.

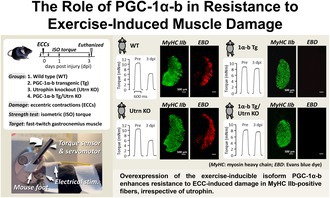

## 1 INTRODUCTION

Eccentric contractions (ECCs) are considered more effective in promoting skeletal muscle hypertrophy compared to other contraction modes (Ashida et al., 2018). However, ECCs are also well known for their potential to induce muscle damage, and excessive ECC loading can lead to prolonged muscle weakness lasting from several days to weeks (Ashida et al., 2021; Lavender & Nosaka, 2006; Warren et al., 2001; Yamada et al., 2018). The appearance of creatine kinase (CK), an intracellular muscle enzyme, in the blood following ECCs supports the membrane tear hypothesis, which proposes that the increased mechanical stress during ECCs directly damages the muscle fiber membrane (Moens et al., 1993; Petrof et al., 1993). Nevertheless, histological analyses have shown that membrane disruption is rarely observed immediately after ECC loading (Jones et al., 1986; Komulainen et al., 1994; Yamada et al., 2018), and blood CK levels do not consistently correlate with the extent of muscle damage (Fridén & Lieber, 2001), casting doubt on this hypothesis (Allen et al., 2016). More recent studies highlight the role of elevated intracellular Ca^2+^ concentrations, mediated by stretch‐activated channels in the sarcolemma (Zhang et al., 2012) and/or Ca^2+^ release channels in the sarcoplasmic reticulum (Tabuchi et al., 2022), as key triggers of ECC‐induced muscle damage.

Previous studies have shown that susceptibility to ECC‐induced damage varies significantly depending on muscle fiber type (Choi & Widrick, 2010; Jones et al., 1986; Lieber & Fridén, 1988). Muscle fiber type is defined by the expression of specific myosin heavy chain (MyHC) isoforms and associated metabolic characteristics. Although some earlier studies referred to fast‐twitch glycolytic type IIb fibers in humans, it is now well established that human skeletal muscle does not express the MyHC IIb isoform. Instead, fibers previously classified as type IIb by ATPase histochemistry actually express MyHC IIx and should be considered type IIx (also known as type IId) fibers (Smerdu et al., 1994). In contrast, rodents express distinct fast‐twitch glycolytic type IIb fibers. Type IIb fibers have been reported to be more susceptible to ECC‐induced damage than other fiber types in experimental animals (Lieber & Fridén, 1988), while in humans, type IIx fibers are considered the most vulnerable (Choi & Widrick, 2010; Jones et al., 1986). However, the mechanisms underlying fiber type‐specific susceptibility to ECC‐induced damage remain unclear.

Peroxisome proliferator‐activated receptor gamma coactivator‐1alpha (PGC‐1α) is a transcription factor that regulates oxidative capacity, including mitochondrial quantity and function, in skeletal muscle. PGC‐1α exists in multiple isoforms, with the major isoform being PGC‐1α1, also referred to as PGC‐1α‐a (Jannig et al., 2022; Ruas et al., 2012). The expression of PGC‐1α1/‐a is lower in type IIb fibers, which have limited oxidative capacity, compared to type I fibers (Lin et al., 2002). In mouse models of Duchenne muscular dystrophy (DMD), the absence of dystrophin greatly increases susceptibility to ECCs‐induced damage (Blaauw et al., 2010; Petrof et al., 1993). Interestingly, overexpression of PGC‐1α1/‐a in DMD model animals has been reported to improve resistance to ECC‐induced damage (Handschin et al., 2007; Hollinger et al., 2013). Although these studies did not distinguish between fiber types, type IIb fibers are known to be especially vulnerable in dystrophin‐deficient muscle (Webster et al., 1988), suggesting that increased expression of PGC‐1α1/‐a may enhance damage resistance in these fibers.

Compared to the PGC‐1α1/‐a isoform, PGC‐1α‐b mRNA is transcribed from an alternative exon 1 of the PGC‐1α gene (Yoshioka et al., 2009). This isoform has been shown to be highly responsive to exercise (Nomura et al., 2024; Tadaishi, Miura, Kai, Kano, et al., 2011; Tadaishi, Miura, Kai, Kawasaki, et al., 2011). Intriguingly, several studies have reported that prior endurance training enhances resistance to ECC‐induced muscle damage (Gosselin, 2000; Hughes & Gosselin, 2002). Based on these findings, it is conceivable that oxidative fibers, including those in muscle fibers that shift toward a more oxidative phenotype following endurance training, exhibit higher expression of the PGC‐1α‐b isoform, which in turn contributes to their increased resistance to damage.

PGC‐1α1/‐a increases the expression of utrophin (Utrn), a costameric protein, resulting in lower Utrn levels in fast‐twitch fibers compared to slow‐twitch fibers (Angus et al., 2005). With regard to the mechanism by which PGC‐1α1/‐a confers to damage in dystrophin‐deficient muscles, several studies suggested that elevated Utrn expression plays a role (Handschin et al., 2007; Hollinger et al., 2013; Selsby et al., 2012). However, recent findings (Lindsay et al., 2019) indicate that utrophin does not significantly contribute to sarcolemmal stability in dystrophin‐intact muscle. This raises the possibility that utrophin may not play a major structural role in ECC‐induced damage resistance in normal muscle, and that its relevance may be limited to dystrophin‐deficient conditions. Nevertheless, PGC‐1α‐b may induce a broad transcriptional program that includes utrophin upregulation, and it remains unclear whether this contributes functionally to damage resistance in wild‐type (WT) muscle.

In this study, therefore, we aim to test two hypotheses: first, that skeletal muscle‐specific overexpression of PGC‐1α‐b enhances the ability of fast‐twitch, MyHC IIb‐positive fibers to resist ECC‐induced structural damage and preserve contractile function; and second, that this protective effect is mediated through increased Utrn expression, as assessed by examining the impact of PGC‐1α‐b overexpression on damage resistance in Utrn‐deficient mice.

## 2 MATERIALS AND METHODS

### 2.1 Ethical approval

Male C57BL/6N WT mice were purchased from Japan SLC, Inc. (Shizuoka, Japan). Transgenic mice overexpressing the PGC‐1α‐b in the skeletal muscle (PGC‐1α‐b Tg, C57BL/6N background) were provided by the National Institute of Biomedical Innovation (Miura et al., 2008; Tadaishi, Miura, Kai, Kano, et al., 2011). Utrn knockout (Utrn KO, C57BL/6J background) mice were kindly provided by Dr. Kay Davies (University of Oxford) (Deconinck et al., 1997). Heterozygotes PGC‐1α‐b Tg mice were crossbred with homozygous Utrn KO mice, and the offspring were backcrossed into the Utrn KO background. Both Utrn KO and heterozygous PGC‐1α‐b Tg/homozygous Utrn KO (PGC‐1α‐b Tg/Utrn KO) mice were obtained. Male and female offspring aged 12–18 weeks were used in experiments. All mice were maintained on standard laboratory rodent chow CRF‐1 (Oriental Yeast Co., Ltd., Tokyo, Japan) and water under a 12 h light–dark cycle.

All mouse experiments were performed at Sapporo Medical University (Sapporo, Japan) in accordance with protocols approved by the Committee on Animal Experiments of Sapporo Medical University (No. 20‐108_21‐060_23‐021_23‐049_25‐039). Animal care was in accordance with institutional guidelines. For in vivo muscle experiments, mice were anesthetized with 2% inhaled isoflurane to reach a stable anesthetic plane with a consistent breathing rate and no response to toe pinch. At the end of the experiment, mice were euthanized by rapid cervical dislocation under isoflurane anesthesia, and muscles were subsequently isolated. A total of 48 mice were used.

### 2.2 Experimental procedures

To investigate the roles of PGC‐1α‐b and Utrn in resistance to ECC‐induced muscle damage, we examined whether skeletal muscle‐specific overexpression of PGC‐1α‐b enhances damage resistance in WT and Utrn KO mice. Accordingly, WT (male, *n* = 12), PGC‐1α‐b Tg (male, *n* = 12), Utrn KO (male, *n* = 10; female, *n* = 2), and PGC‐1α‐b Tg/Utrn KO mice (male, *n* = 5; female, *n* = 7) were subjected to 100 repeated ECCs.

The left plantar flexor muscles were stimulated supramaximally (45 V) via a pair of surface electrodes every 4 s under isoflurane anesthesia (WT + ECC, PGC‐1α‐b Tg + ECC, Utrn KO + ECC, and PGC‐1α‐b Tg/Utrn KO + ECC groups). ECCs consisted of forced dorsiflexion from 0° to 40° at a velocity of 150°/s, combined with electrical stimulation (0.5 ms monophasic rectangular pulses at 50 Hz), as previously described (Ashida et al., 2021, 2022; Yamada et al., 2018). The right contralateral muscles served as controls (WT, PGC‐1α‐b Tg, Utrn KO, and PGC‐1α‐b Tg/Utrn KO groups). In vivo isometric torque was measured at stimulation frequencies of 30, 50, and 100 Hz immediately before (Pre), immediately after (0 days post‐injury; 0 dpi), and at 1 and 3 dpi following 100 repeated ECCs.

At both 0 and 3 dpi, mice were euthanized by cervical dislocation under isoflurane anesthesia, and the fast‐twitch gastrocnemius (GST) and plantaris (PLA) muscles, as well as the slow‐twitch soleus muscles, were collected from each group (*n* = 6 per group). The PLA and soleus muscles were snap‐frozen in liquid nitrogen and stored at −80°C for later analysis (i.e., citrate synthase (CS) activity or real‐time PCR). For histological analysis, the mid‐belly of the medial GST muscles was frozen in pre‐cooled isopentane and stored at −80°C. The remaining portions of the GST muscles were quickly minced with scissors on ice (4°C) and stored at −80°C for subsequent biochemical analyses (i.e., real‐time PCR, immunoblots, and MyHC isoforms).

### 2.3 Quantitative real‐time PCR

Real‐time PCR was used to quantify mRNA levels for total PGC‐1α, PGC‐1α1/‐a, PGC‐1α‐b, PGC‐1α‐c, PGC‐1α2, PGC‐1α3, PGC‐1α4, and PGC‐1β in frozen GST muscle tissue. Briefly, total RNA was extracted using TORIZOL reagent (#15596018, Invitrogen, Carlsbad, CA), and RNA purity and concentration were assessed by measuring absorbance at 260 and 280 nm with a Nanodrop Light spectrophotometer (Thermo Scientific, Waltham, MA). cDNA was synthesized from total RNA using the Prime Script RT Reagent Kit (#RR037A, Takara, Japan). Quantitative real‐time PCR was performed with the StepOne™ Real‐Time PCR System (Applied Biosystems, Foster City, CA) using the SYBR Green PCR Master Mix protocol (#4367659, Applied Biosystems, Foster City, CA). The specific primers used are listed in Table 1. Oligonucleotides were purchased from FASMAC Co. Ltd. (Kanagawa, Japan). All reactions were performed in duplicate. Relative mRNA expression levels were calculated using the comparative threshold cycle method (ΔΔCT), and values were normalized to 36B4 mRNA expression.

**TABLE 1:** Primers used for quantitative real‐time PCR.

Gene	References	Sequence
Total PGC‐1α	Ruas et al. (2012)	F: TGATGTGAATGACTTGGA ACAGACA
		R: GCTCATTGTTGTACTGGTTGGATATG
PGC‐1α‐1/‐a	Ruas et al. (2012)	F: GGACATGTGCAGCCAAGACTCT
		R: CACTTCAATCCACCCAGAAAGCT
PGC‐1α‐2	Ruas et al. (2012)	F: CCACCAGAATGAGTGACATGGA
		R: GTTCAGCAAGATCTGGGCAAA
PGC‐1α‐3	Ruas et al. (2012)	F: AAGTGAGTAACCGGAGGCATTC
		R: TTCAGGAAGATCTGGGCAAAGA
PGC‐1α‐4	Ruas et al. (2012)	F: TCACACCAAACCCACAGAAA
		R: CTGGAAGATATGGCACAT
PGC‐1α‐b	Tadaishi, Miura, Kai, Kawasaki, et al. (2011)	F: GACATGGATGTTGGGATTGTCA
		R: ACCAACCAGAGCAGCACATTT
PGC‐1α‐c	Tadaishi, Miura, Kai, Kawasaki, et al. (2011)	F: TGAGTAACCGGAGGCATTCTCT
		R: TGAGGACCGCTAGCAAGTTTG
PGC‐1β	Hatazawa et al. (2016)	F: CAGCTGTGTGCTGACTTGCC
		R: TCAAAGTCACTGGCGTCCAG
36B4	Tadaishi, Miura, Kai, Kano, et al. (2011)	F: GGCCCTGCACTCTCGCTTTC
		R: TGCCAGGACGCGCTTGT

Abbreviations: F, forward; R, reverse.

### 2.4 Citrate synthase activity

CS activity is frequently used as an indicator of mitochondrial content, although transmission electron microscopy remains the gold standard for accuracy (Larsen et al., 2012). Maximal CS activity was assessed in plantaris muscle homogenates. Briefly, the muscles were homogenized in ice‐cold 100 mM potassium phosphate buffer (100 μL/mg wet wt) and maximal CS activity was measured spectrophotometrically as described previously (Srere, 1969).

### 2.5 Myosin heavy chain isoforms separation

The GST muscles were homogenized in ice‐cold homogenizing buffer (30 μL/mg wet wt) containing (in mM): 10 Tris maleate, 35 NaF, 1 NaVO_4_, 1% Triton X 100 (vol/vol), and one tablet of protease inhibitor cocktail (#11697498001, Roche, Switzerland) per 50 mL. Protein concentration was determined using Bradford assay (#5000006, Bio‐Rad, Hercules, CA) (Bradford, 1976). Aliquots of the whole muscle homogenates (5 μg) were mixed with SDS sample buffer containing (in mM): 62.5 Tris/HCl, 2% SDS (wt/vol), 10% glycerol (vol/vol), 5% 2‐mercaptoethanol (vol/vol), and 0.02% bromophenol blue (wt/vol). Proteins were separated using a 6.8% polyacrylamide slab gel, as described previously (Yamada et al., 2004). Electrophoresis was performed at 160 V for 24 h at 4°C. Gels were stained with Coomassie brilliant blue, and band intensities were analyzed densitometrically using ImageJ software.

### 2.6 Immunoblots

Immunoblotting was performed using the following primary antibodies: anti‐total OXPHOS rodent WB antibody cocktail (1:1000, ab110413, Abcam, Cambridge, UK), anti‐junctophilin (JP) 1 (1:10,000, 40‐5100, Thermo Fisher, Waltham, MA), anti‐SH3 and cysteine‐rich domain (STAC) 3 (1:10,000, 20392‐1‐AP, Proteintech, Rosemont, IL), anti‐calpain‐1 (1:1000, C0355, Sigma‐Aldrich, St. Louis, MO), anti‐utrophin (1:1000, kindly provided by Dr. Michihiro Imamura, National Center of Neurology and Psychiatry [NCNP], Japan) (Imamura & Ozawa, 1998), anti‐dystrophin (1:500, ab15277, Abcam, Cambridge, UK), anti‐integrin α7B (1:1000, kindly provided by Dr. Eva Engvall, Burnham Institute, La Jolla, CA) (Moghadaszadeh et al., 2003), anti‐integrin β1D (1:1000, kindly provided by Dr. Eva Engvall, Burnham Institute, La Jolla, CA) (Moghadaszadeh et al., 2003), anti‐α‐sarcoglycan (1:1000, kindly provided by Dr. Michihiro Imamura, NCNP, Japan) (Araishi et al., 1999), and anti‐β‐dystroglycan (1:1000, kindly provided by Dr. Michihiro Imamura, NCNP, Japan) (Imamura et al., 2000).

Aliquots (5 μg) of GST muscle homogenates (see MyHC isoforms separation) were diluted in SDS sample buffer and loaded onto 4%–15% Criterion Stain‐Free gel (#4568086, BioRad, Hercules, CA). Gels were imaged using Bio‐Rad Stain‐Free imager, and proteins were transferred to polyvinylidine fluoride membranes. Membranes were blocked in 3% (wt/vol) nonfat milk in Tris‐buffered saline containing 0.05% (vol/vol) Tween 20, followed by overnight incubation at 4°C with primary antibodies. After washing, membranes were incubated for 1 h at room temperature with secondary antibody (1:10,000, donkey‐anti‐rabbit, #31460, or donkey‐anti‐mouse, #31430, Invitrogen, Carlsbad, CA). Protein bands were visualized using a chemiluminescence substrate (#WBKLS0500, Merck Millipore, Billerica, MA) and imaged with a ChemiDoc MP imaging system (Bio‐Rad). Densitometric analysis was performed using Image Lab Software (Bio‐Rad).

### 2.7 Autolysis of calpain 1

Muscle proteins (20 μg) were separated on a 7% SDS‐polyacrylamide gel and immunoblotting was performed using anti‐calpain 1 antibody (C0355, Sigma‐Aldrich, St. Louis, MO) and donkey‐anti‐mouse (#31430, Invitrogen, Carlsbad, CA) secondary antibody as described by Kanzaki et al. (2019). The amount of autolyzed calpain 1 was expressed as a percentage of total calpain in the same muscle sample. In addition, the expression level of total calpain was quantified using MyHC as a loading control.

### 2.8 Histopathological analyses

Cryostat sections (10 μm) from the mid‐belly of the medial GST muscles were prepared at 0 and 3 dpi and stained with hematoxylin and eosin (H&E). For Evans Blue dye (EBD) quantification, a 1% (wt/vol) EBD solution (1 mg/10 g body wt) was intraperitoneally injected under isoflurane anesthesia 9 h before euthanasia (Himori et al., 2021). Whole‐section images were captured using a BZ‐X700 microscope (KEYENCE, Osaka, Japan) with a 4× objective, stitched together using BZ‐X700 analyzer software (KEYENCE), and exported as TIFF files. To determine EBD‐positive areas, fluorescence intensity thresholds were established based on WT muscle sections that did not undergo eccentric contraction, which served as negative controls. Only regions with fluorescence intensity exceeding the baseline threshold were considered EBD‐positive. The EBD‐positive area was quantified as a percentage of the total cross‐sectional muscle area using the same software.

### 2.9 Immunohistochemical analyses

Cryosections (10 μm thick) of the GST muscle were prepared at 3 dpi. Sections were air‐dried for 30 min, then rinsed in cold PBS followed by a rinse in PBS at room temperature. Then, the sections were fixed in 4% paraformaldehyde for 3 min and washed for 2 min in cold PBS followed by 3 min in PBS at room temperature. Subsequently, the sections were blocked and permeabilized in a blocking solution containing 10% normal goat serum, 1% Triton X‐100, and 2% bovine serum albumin (BSA) in PBS. Primary antibodies were diluted in PBS containing 5% normal goat serum, 0.3% Triton X‐100, and 2% BSA, then applied to the slides and incubated overnight at 4°C. The following primary antibodies from the Developmental Studies Hybridoma Bank were used: anti‐MyHC type IIb (BF‐F3; 1:150), anti‐MyHC type IIa (SC‐71; 1:50), and anti‐MyHC type I (BA‐D5; 1:50). Following primary antibody incubation, sections were washed in PBS for 20 min. Secondary antibodies were diluted 1:200 in PBS containing 5% normal goat serum, 0.1% Triton X‐100, and 2% BSA, then applied to the slides and incubated for 1 h at room temperature. The following secondary antibodies were used: goat anti‐mouse IgG2b Alexa Fluor™ 546 (#A‐21143), goat anti‐mouse IgG1 Alexa Fluor™ 350 (#A‐21120), and goat anti‐mouse IgM Alexa Fluor™ 488 (#A‐21042) (Thermo Fisher Scientific, Waltham, MA). After a final 20‐min PBS wash, the sections were mounted using Hardset Mounting Medium (H‐1700, VECTASHIELD, Vector Laboratories, Burlingame, CA). Images were acquired using a fluorescence microscope (BZ‐X700, KEYENCE, Osaka, Japan) equipped with 4× and 20× objective lenses and captured using the BZ‐X700 analyzer software (KEYENCE). To ensure objectivity, all image acquisition and analysis were performed in a blinded manner. Subsequently, the proportion of each muscle fiber type among all EBD‐positive fibers was calculated in both WT+ECC and Utrn KO+ECC groups. In addition, the overall distribution of MyHC I, IIa, and IIb‐positive fibers was quantified across all four experimental groups to assess fiber‐type composition and its potential relationship with susceptibility to ECC‐induced damage. It should be noted that fibers positive for MyHC IIb may co‐express other isoforms such as MyHC IIx and are therefore referred to as “MyHC IIb‐positive fibers” throughout the manuscript.

### 2.10 Statistics

Data are presented as mean ± SD. Data normality was examined using the Shapiro–Wilk test. An unpaired *t*‐test was used to assess statistical differences in the mRNA expression of PGC‐1α isoforms (i.e., PGC‐1α1/‐a, ‐1α2, ‐1α3, ‐1α4, ‐1β), CS activity, and OXPHOS protein levels (i.e., CI, CII, CIII, and CV subunits) in the GST muscle between WT and PGC‐1α‐b Tg mice. Differences in PGC‐1α‐b expression between the GST and soleus muscles were also examined within WT mice. In contrast, the Mann–Whitney rank‐sum test was applied to non‐normally distributed variables, including mRNA levels of total PGC‐1α, PGC‐1α‐b, and PGC‐1α‐C, as well as the CIV subunit.

A one‐way ANOVA was conducted to evaluate MyHC IIb and MyHC IIx/a levels, isometric specific torque, percentage of torque at 30 Hz and 50 Hz relative to maximum torque at 100 Hz across the four groups (WT, PGC‐1α‐b Tg, Utrn KO, and PGC‐1α‐b Tg/Utrn KO group), whereas MyHC I, EBD positive area, total weight of plantar flexor muscles, specific isometric torque at 30 Hz, and specific eccentric torque were analyzed using the Kruskal–Wallis one‐way ANOVA on ranks. Torque drop during ECC (genotype × repetitions) and MIT (genotype × dpi) were analyzed by two‐way repeated‐measures ANOVA. Two‐way ANOVA was also used to compare the expression levels of JP1, STAC3, the proportion of calpain‐1 autolysis, Utrn, dystrophin, integrin α7B, integrin β1D, α‐sarcoglycan, and β‐dystroglycan (genotype × ECC). When ANOVA indicated significance, Tukey's post hoc test was performed. A *p* value <0.05 was considered statistically significant.

A power analysis was conducted based on the assumption that PGC‐1α‐b overexpression induces a 35% ± 15% change in physiological measurements relative to the control (Du et al., 2025). With a power of 0.80 and an alpha of 0.05, the required sample size was calculated to be six. Accordingly, six animals were used per group in all experiments, although some analyses (i.e., mRNA expression of PGC‐1α isoforms) were performed with *n* = 5–6. Statistical analyses were performed using SigmaPlot (version 13, Systat Software, Inc.).

## 3 RESULTS

### 3.1 Overexpression of PGC‐1α‐b enhances mitochondrial biogenesis and respiratory complex expression in skeletal muscle

**FIGURE 1:**
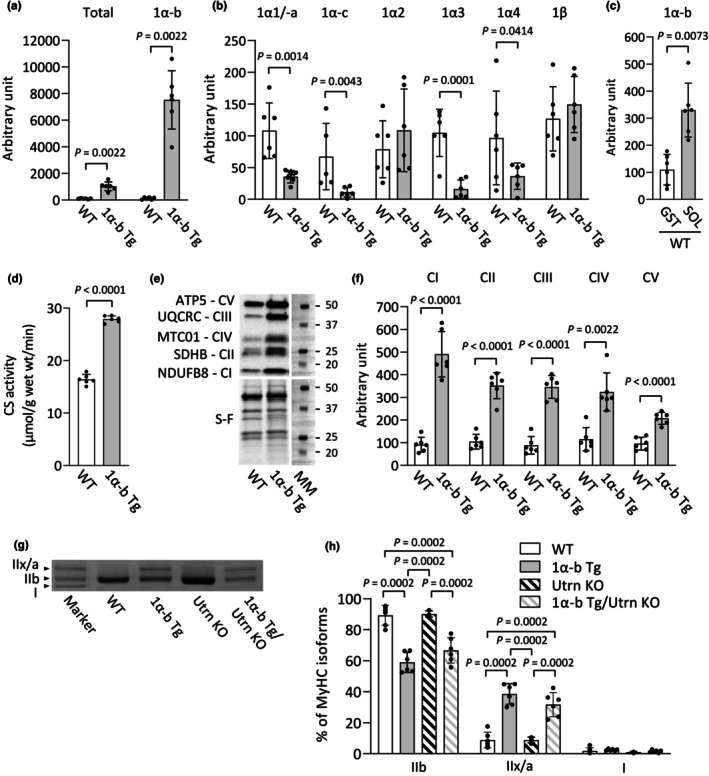
Overexpression of PGC‐1α‐b enhances mitochondrial biogenesis and respiratory complex expression in skeletal muscle. (a) mRNA levels of total PGC‐1α and PGC‐1α‐b, and (b) mRNA levels of PGC‐1α1/‐a, PGC‐1α‐c, PGC‐1α2, PGC‐1α3, PGC‐1α4, and PGC‐1β in the gastrocnemius (GST) muscle from wild‐type (WT) and PGC‐1α‐b transgenic (Tg) mice. (c) mRNA levels of PGC‐1α‐b in the GST and soleus muscles in WT mice. (d) Citrate synthase activity in plantaris muscles. (e) Representative Stain‐Free (S‐F) images and western blots illustrating the protein levels of mitochondrial respiratory chain subunits: Complex I (CI) (NDUFB8), CII (SDHB), CIII (UQCRC), CIV (MTC01), CV (ATP5) in GST muscles. Molecular weight markers (MM). (f) Quantification of CI‐CV subunits normalized to total protein in S‐F images. Data show means ± SD for 5–6 muscles per group. Student's paired *t*‐test was used, except for mRNA levels of total PGC‐1α, PGC‐1α‐b, and PGC‐1α‐C, and the CIV subunit, which were analyzed using the Mann–Whitney rank‐sum test. *p < 0.05 versus WT. (g) Representative blots showing electrophoretically separated myosin heavy chain (MyHC) isoforms in the GST muscles from WT, PGC‐1α‐b Tg, utrophin knockout (Utrn KO), and PGC‐1α‐b Tg/Utrn KO mice. (h) Distribution of MyHC isoforms. I, slow isoform and IIx/a and IIb, fast isoforms. Data show means ± SD for 6 muscles per group. One‐way ANOVA was performed followed by Tukey's post hoc test, except for the percentage of MyHC I, where a Kruskal–Wallis one‐way ANOVA was used on ranks. Statistical significance was set at *p* < 0.05.

To specifically evaluate the molecular and metabolic effects of PGC‐1α‐b overexpression in skeletal muscle, independent of potential confounding influences from Utrn deficiency, initial analyses were conducted by comparing PGC‐1α‐b transgenic (Tg) mice with wild‐type (WT) controls. This approach allowed us to isolate the impact of PGC‐1α‐b on muscle phenotype and mitochondrial regulation without the added complexity of the Utrn KO background. In the gastrocnemius (GST) muscles of PGC‐1α‐b Tg mice, the mRNA levels of total PGC‐1α and PGC‐1α‐b were significantly higher than in WT mice (total PGC‐1α, *p* = 0.0022; PGC‐1α‐b, *p* = 0.0022) (Figure 1a). It should be noted that PGC‐1α‐b is an exercise‐inducible isoform, and its expression should be relatively low under resting conditions. Therefore, although PGC‐1α‐b is overexpressed in the Tg model, the increase may not be fully reflected in the total PGC‐1α measurement due to the low baseline expression in WT mice. In contrast, mRNA levels of PGC‐1α1/‐a, PGC‐1α‐c, PGC‐1α3, and PGC‐1α4 were lower in PGC‐1α‐b Tg mice compared to WT mice (PGC‐1α1/‐a, *p* = 0.0014; PGC‐1α‐c, *p* = 0.0043; PGC‐1α3, *p* = 0.0001; PGC‐1α4, *p* = 0.0414) (Figure 1b). There were no significant differences in the mRNA levels of PGC‐1α2 and PGC‐1β mRNA between the two groups. Furthermore, in WT mice, the mRNA expression level of PGC‐1α‐b was higher in the slow‐twitch soleus muscle than in the fast‐twitch GST muscle (*p* = 0.0073) (Figure 1c).

CS activity measured in the plantaris muscle, as well as the expression levels of mitochondrial respiratory chain complexes I–V assessed in the GST, were higher in PGC‐1α‐b Tg mice compared to WT mice (CS activity, *p* < 0.0001; CI, *p* < 0.0001; CII, *p* < 0.0001; CIII, *p* < 0.0001; CIV, *p* = 0.0022; CV, *p* < 0.0001) (Figure 1d–f). Conversely, there was no difference in MyHC isoform expression in the GST muscle between WT and Utrn KO mice, as well as between PGC‐1α‐b Tg and PGC‐1α‐b Tg/Utrn KO mice (Figure 1g,h). The proportion of the MyHC IIb isoform was significantly lower in the GST muscles of PGC‐1α‐b Tg (*p* = 0.0002 vs. WT, *p* = 0.0002 vs. Utrn KO) and PGC‐1α‐b Tg/Utrn KO (*p* = 0.0002 vs. WT, *p* = 0.0002 vs. Utrn KO) mice compared to WT and Utrn KO mice. On the other hand, the proportion of MyHC IIx/a isoform was significantly higher in PGC‐1α‐b Tg (*p* = 0.0002 vs. WT, *p* = 0.0002 vs. Utrn KO) and PGC‐1α‐b Tg/Utrn KO (*p* = 0.0002 vs. WT, *p* = 0.0002 vs. Utrn KO) mice than in WT and Utrn KO mice. There were no significant differences in the proportion of the MyHC I isoform among the groups.

### 3.2 Overexpression of PGC‐1α‐b attenuates torque drop during eccentric contractions in both normal and utrophin knockout mice

**FIGURE 2:**
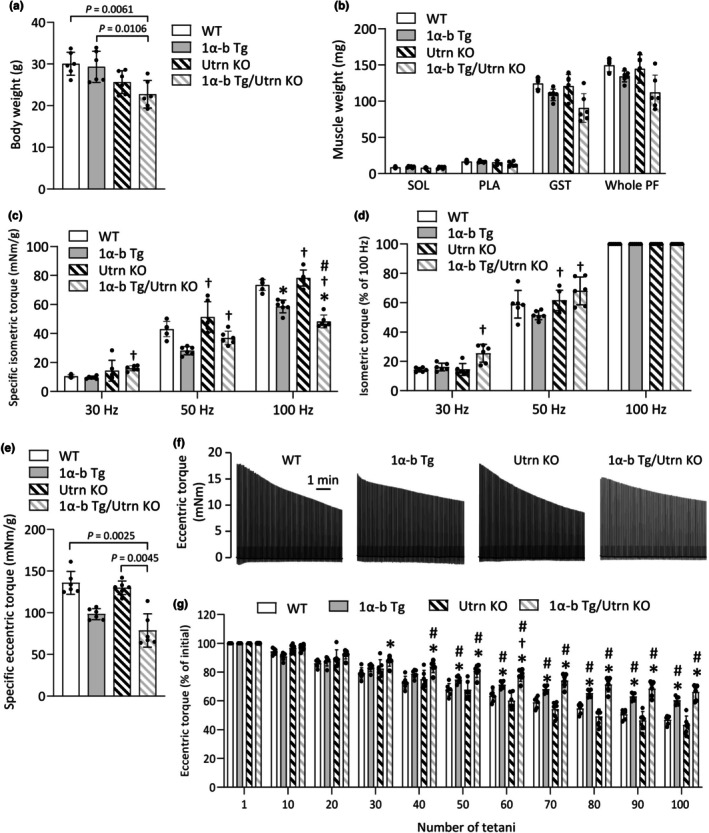
Overexpression of PGC‐1α‐b attenuates torque drop during eccentric contractions in both normal and utrophin knockout mice. Body weight (a) and muscle weights of the soleus (SOL), plantaris (PLA), gastrocnemius (GST), and whole plantar flexor (PF) muscles (b) in wild‐type (WT), PGC‐1α‐b transgenic (PGC‐1α‐b Tg), utrophin knockout (Utrn KO), and PGC‐1α‐b Tg/Utrn KO mice. Absolute isometric torque normalized to the weight of whole PF (specific isometric torque) (c). Percentage of force generated at stimulation frequencies of 30 Hz and 50 Hz, relative to the maximum isometric torque measured at 100 Hz stimulation (d). Specific eccentric torque (**e**). Data show means ± SD for 6 muscles per group. Statistical analysis was performed using one‐way ANOVA followed by Tukey's post hoc test, except for whole PF weight, specific isometric torque at 30 Hz, and specific eccentric torque, which were analyzed using Kruskal‐Wallis one‐way ANOVA on ranks. *p < 0.05 versus WT, ^†^ *p* < 0.05 versus PGC1α‐b Tg, ^#^ *p* < 0.05 versus Utrn KO. Typical torque traces during eccentric contractions (ECCs) (f). Mean (±SD) relative torque during ECCs (g). The torque in the first tetanus was set to 100% for each muscle. Data show means ± SD for 6 muscles per group. Two‐way repeated‐measures ANOVA was performed, followed by Tukey's post hoc test. *p < 0.05 versus WT, ^†^ *p* < 0.05 versus PGC1α‐b Tg, ^#^ *p* < 0.05 versus Utrn KO.

Compared to the WT group, the PGC‐1α‐b Tg/Utrn KO group exhibited significantly lower body weight (*p* = 0.0061) (Figure 2a), which is likely attributable to the higher proportion of female mice in this group. In contrast, no significant differences were observed among groups in the muscle weights of the SOL, PLA, GST, or the entire triceps surae muscle group (Figure 2b).

Specific isometric torque, calculated by normalizing absolute isometric torque to the total triceps surae muscle mass, was significantly lower in both the PGC‐1α‐b Tg group (*p* = 0.0003) and the PGC‐1α‐b Tg/Utrn KO group (*p* = 0.0003) at 100 Hz stimulation frequency, which corresponds to the maximum isometric torque (MIT) (Figure 2c). However, torque values at 30 Hz and 50 Hz relative to MIT did not differ significantly between the WT group and either the PGC‐1α‐b Tg or PGC‐1α‐b Tg/Utrn KO groups (Figure 2d).

Specific eccentric torque was lower in the PGC‐1α‐b Tg/Utrn KO group compared to the WT group (*p* = 0.0025) (Figure 2e). A representative torque profile during ECC loading is shown in Figure 2f. Eccentric torque was expressed as a percentage of pre‐injury values to facilitate comparison of relative force loss across groups with differing baseline strength. During repeated eccentric contractions (ECCs), torque values were higher in the PGC‐1α‐b Tg group after the 50th ECC and in the PGC‐1α‐b Tg/Utrn KO group after the 30th ECC, compared to the WT group (Figure 2g).

### 3.3 Overexpression of PGC‐1α‐b improves maximum isometric torque recovery after damaging eccentric contractions in both normal and utrophin knockout mice

**FIGURE 3:**
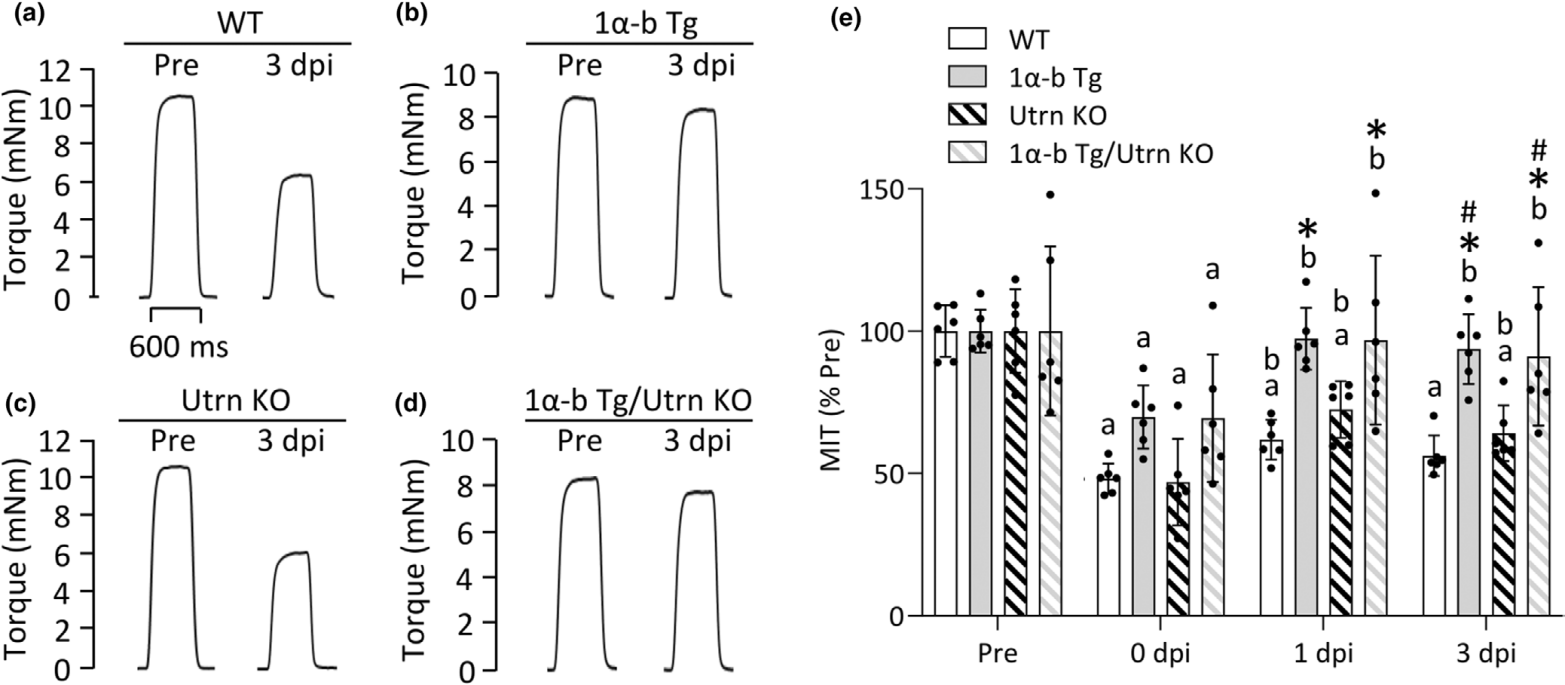
Overexpression of PGC‐1α‐b improves in vivo isometric torque recovery after damaging eccentric contractions in both normal and utrophin knockout mice. Representative traces of maximum isometric torque (MIT) at 100 Hz stimulation frequency of the plantar flexor muscles in wild‐type (WT) (a), PGC‐1α‐b transgenic (PGC‐1α‐b Tg) (b), utrophin knockout (Utrn KO) (c), and PGC‐1α‐b Tg/Utrn KO mice (d) at 3 days post‐injury (dpi) following eccentric contractions (ECCs). MIT was measured before (Pre), immediately after (0 dpi), and at 1 and 3 dpi (e). Data are presented as a percentage of the pre‐ECC value (% Pre) and shown as means ± SD (*n* = 6 per group). Two‐way repeated‐measures ANOVA was performed, followed by Tukey's post hoc test. *p < 0.05 versus WT, ^#^ *p* < 0.05 versus Utrn KO, ^a^ *p* < 0.05 versus Pre, ^b^ *p* < 0.05 versus 0 dpi.

In the WT+ECC group, MIT following ECCs was significantly reduced at 0 dpi (*p* = 0.0002), 1 dpi (*p* = 0.0002), and 3 dpi (*p* = 0.0002) compared to pre‐damage levels (Figure 3). Similar reductions in MIT were observed in the Utrn KO+ECC group at the same time points: 0 dpi (*p* = 0.0002), 1 dpi (*p* = 0.0002), and 3 dpi (*p* = 0.0002). In contrast, the PGC‐1α‐b Tg+ECC group showed significantly higher MIT values than the WT+ECC group at 1 dpi (*p* = 0.0041) and 3 dpi (*p* = 0.0024). Similarly, the PGC‐1α‐b Tg/Utrn KO + ECC group exhibited significantly higher MIT values at 1 dpi (*p* = 0.0047) and 3 dpi (*p* = 0.0047) compared to the WT+ECC group.

### 3.4 Overexpression of PGC‐1α‐b prevents eccentric contraction‐induced sarcolemmal injury in both normal and utrophin knockout mice

**FIGURE 4:**
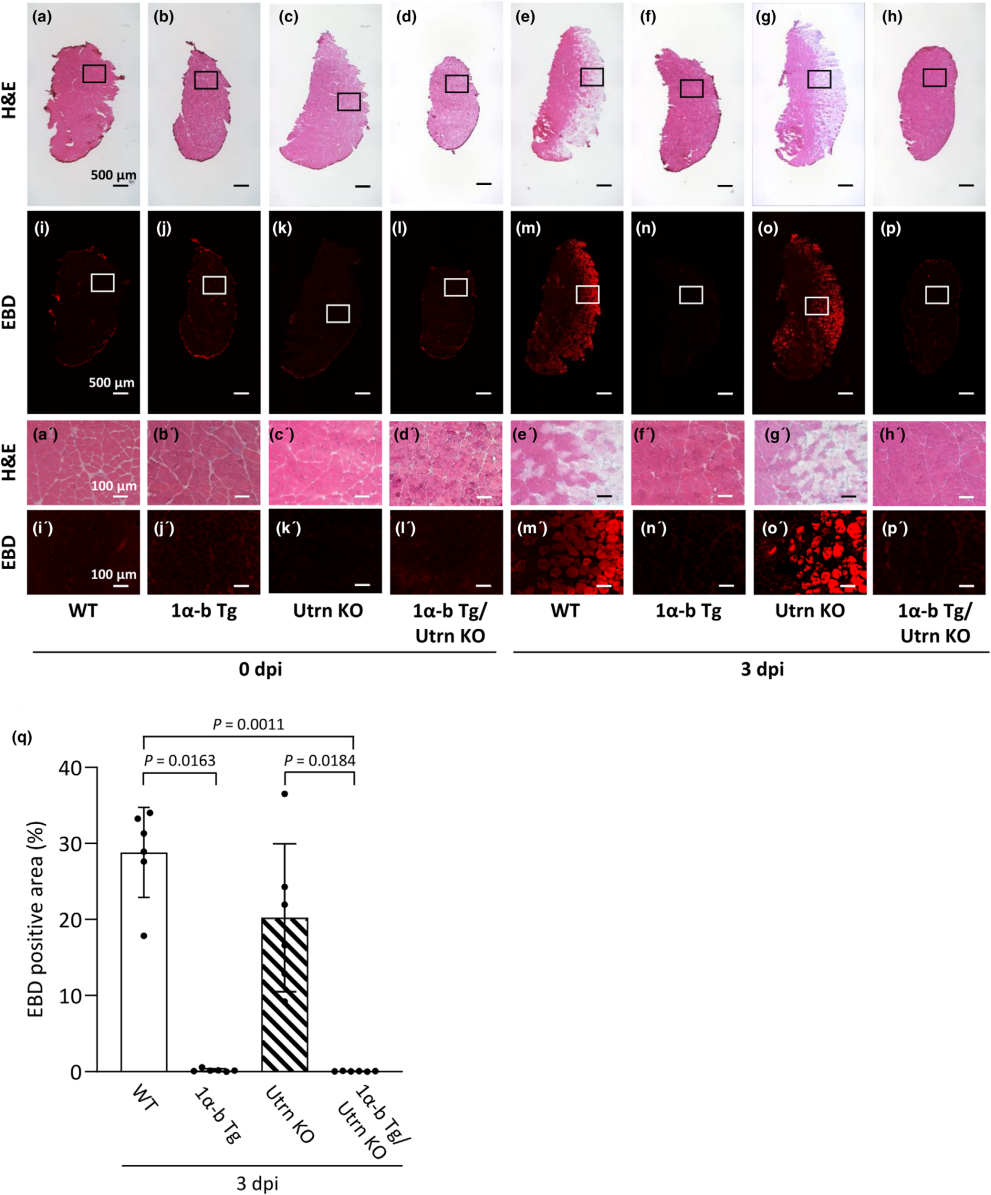
Overexpression of PGC‐1α‐b prevents eccentric contraction‐induced sarcolemmal injury in both normal and utrophin knockout mice. Representative cross‐sectional images stained for Hematoxylin and Eosin (H & E; a–h, a′–h′) and Evans Blue dye (EBD; i–p, i′–p′) of gastrocnemius muscles from wild‐type (WT), PGC‐1α‐b transgenic (Tg), utrophin knockout (Utrn KO), and PGC‐1α‐b Tg/Utrn KO mice 0 and 3 days post injury (dpi) following eccentric contractions. a′–p′ show magnified views of the boxed regions in a–p. Scale bars: 500 μm (a–p) and 100 μm (a′–p′). Quantification of the percentage of EBD‐positive area relative to total muscle cross‐sectional area at 3 dpi (q). Data show means ± SD for 6 muscles per group. Statistical analysis was performed using Kruskal–Wallis one‐way ANOVA on ranks. Statistical significance was set at *p* < 0.05. EBD‐positive fibers were nearly absent at 0 dpi across all groups, resulting in values close to zero.

Representative H&E‐stained images of each group at 0 dpi and 3 dpi are shown in Figure 4a–h and a′–h′, and Evans blue dye (EBD)‐stained tissue sections are shown in Figure 4i–p and i′–p′. At 0 dpi, EBD‐positive fibers were rarely observed in any of the groups. At 3 dpi, a substantial number of EBD‐positive fibers were detected in the WT+ECC and Utrn KO+ECC groups, indicating that ECC induced sarcolemmal damage even in WT muscle (Figure 4q). This is consistent with previous reports showing that fast‐twitch muscles such as the GST are highly susceptible to ECC‐induced injury, regardless of dystrophin or utrophin status (Ashida et al., 2021; Tokuda et al., 2025; Yamada et al., 2018). Importantly, only a few EBD‐positive fibers were observed at 3 dpi in the PGC‐1α‐b Tg+ECC group compared with the WT+ECC group (*p* = 0.0163 vs. WT+ECC). Furthermore, the number of EBD‐positive fibers was significantly lower in the PGC‐1α‐b Tg/Utrn KO+ECC groups compared with both WT+ECC and Utrn KO+ECC groups (*p* = 0.0011 vs. WT+ECC, *p* = 0.0184 vs. Utrn KO+ECC), highlighting the protective effect of PGC‐1α‐b overexpression against ECC‐induced muscle damage.

**FIGURE 5:**
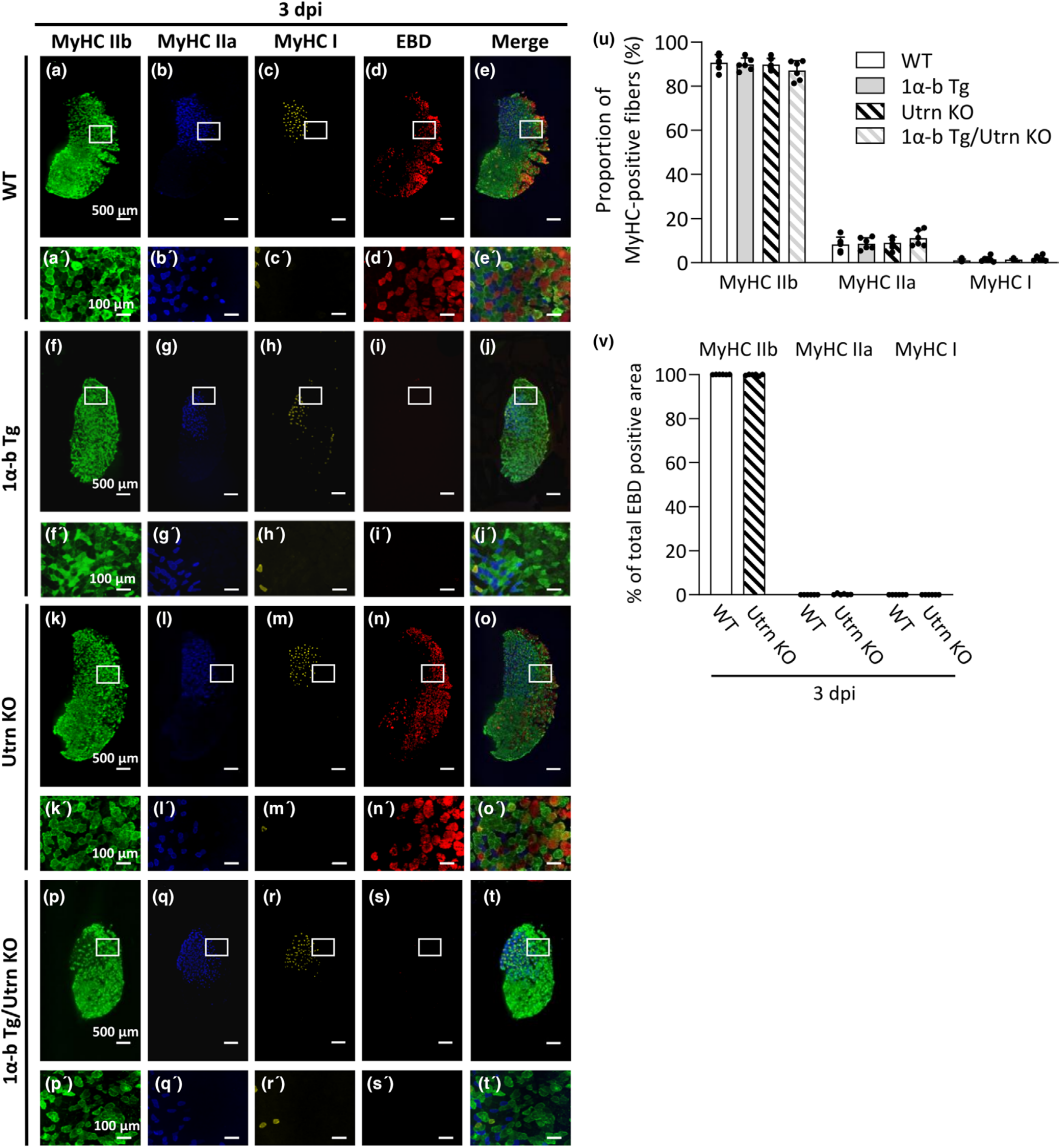
Overexpression of PGC‐1α‐b prevents eccentric contraction‐induced sarcolemmal injury in myosin heavy chain IIb‐positive fibers. Representative cross‐sectional images of gastrocnemius muscles from wild‐type (WT), PGC‐1α‐b transgenic (Tg), utrophin knockout (Utrn KO), and PGC‐1α‐b Tg/Utrn KO mice stained for myosin heavy chain (MyHC) IIb (green; a, a′, f, f′, k, k′, p, and p′), MyHC IIa (blue; b, b′, g, g′, l, l′, q, and q′), MyHC I (yellow; c, c′, h, h′, m, m′, r, and r′), Evans blue dye (red; d, d′, i, i′, n, n′, s, and s′) and merged images (e, e′, j, j′, o, o′, t, and t′) at 3 days post‐injury (3 dpi) following eccentric contractions. a′–t′ show magnified views of the boxed regions in a–t. Scale bars 500 μm (a–t) and 100 μm (a′–t′). Proportion of MyHC IIb‐, MyHC IIa‐, and MyHC I‐positive fibers among total muscle fibers in each group (u). Distribution of each muscle fiber type among all EBD‐positive fibers in the WT+ECC and Utrn KO+ECC groups (v).

Figure 5 shows representative immunostaining images at 3 dpi for MyHC IIb, MyHC IIa, and MyHC I, as well as corresponding EBD‐stained images and merged images. These results indicate that the proportion of muscle fibers expressing MyHC IIb, MyHC IIa, and MyHC I in PGC‐1α‐b Tg and PGC‐1α‐b Tg/Utrn KO mice is largely comparable to that in WT and Utrn KO mice (Figure 5u). In contrast, SDS‐PAGE analysis of MyHC isoforms revealed an increased proportion of MyHC IIx/a in the PGC‐1α‐b Tg and PGC‐1α‐b Tg/Utrn KO groups (see Figure 1h). These findings suggest that while the levels of oxidative fiber types such as MyHC IIa and MyHC I remain unchanged, the expression of MyHC IIx is specifically upregulated in these mice.

On the other hand, in the WT+ECC and Utrn KO+ECC groups, EBD‐positive fibers observed at 3 dpi were almost exclusively MyHC IIb‐positive fibers, with very few EBD‐positive fibers detected in MyHC IIa‐ or MyHC I‐positive fibers (Figure 5v). Strikingly, EBD‐positive fibers were rarely observed even among MyHC IIb‐positive fibers in the PGC‐1α‐b Tg+ECC and PGC‐1α‐b Tg/Utrn KO+ECC groups.

### 3.5 Overexpression of PGC‐1α‐b prevents eccentric contraction‐induced degradation of excitation‐contraction coupling proteins in both normal and utrophin knockout mice

**FIGURE 6:**
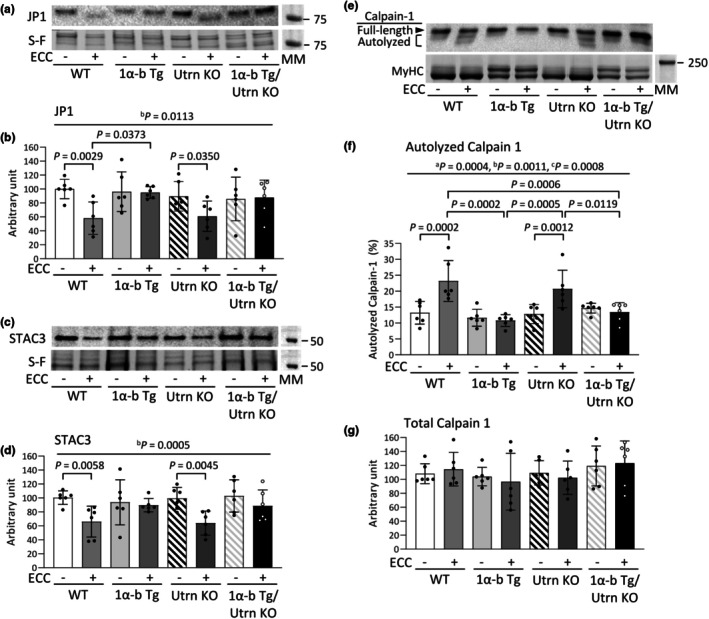
Overexpression of PGC‐1α‐b prevents eccentric contraction‐induced calpain‐1 activation and protein degradation in both normal and utrophin knockout mice. Representative Stain‐Free (S‐F) images and western blots illustrating the levels of junctophilin (JP) 1 (a) and SH3 and cysteine‐rich domain (STAC) 3 (c), and calpain‐1 autolysis (e) in the gastrocnemius muscles of wild‐type (WT), PGC‐1α‐b transgenic (Tg), utrophin knockout (Utrn KO), and PGC‐1α‐b Tg/Utrn KO mice at 3 days post‐injury (3 dpi) following eccentric contractions (ECCs). The levels of JP1 (b) and STAC3 (d) were normalized to total protein from the corresponding S‐F images. The autolyzed form of calpain‐1 is expressed as a percentage of the total calpain‐1 in each muscle sample (f). Total calpain‐1 levels normalized to myosin heavy chain (MyHC) content (g). Molecular weight markers (MM). Data show means ± SD for 6 muscles per group. Two‐way ANOVA was performed, followed by Tukey's post hoc test. Statistical significance was set at *p* < 0.05: Main effect of ^a^genotype and ^b^ECCs; ^c^interaction of genotype and ECCs.

Prolonged force depression after ECC has been reported to involve the degradation of excitation–contraction (E‐C) coupling proteins, such as junctophilin 1 (JP1) and SH3 and cysteine‐rich domain 3 (STAC3), through activation of the Ca^2+^‐dependent protease calpain‐1 (Ashida et al., 2021). Therefore, we investigated whether overexpression of PGC‐1α‐b prevents these degenerative changes induced by ECC. At 3 dpi, compared to the WT group, the WT+ECC group exhibited significantly reduced expression levels of both JP1 and STAC3 ([JP1] WT vs. WT+ECC, *p* = 0.0029; [STAC3] WT vs. WT+ECC, *p* = 0.0058) (Figure 6a–d). Similarly, in the Utrn KO+ECC group, the expression levels of JP1 and STAC3 were significantly decreased compared to the Utrn KO group ([JP1] Utrn KO vs. Utrn KO+ECC, *p* = 0.0350; [STAC3] Utrn KO vs. Utrn KO+ECC, *p* = 0.0045). In contrast, in both PGC‐1α‐b Tg and PGC‐1α‐b Tg/Utrn KO mice, there were no significant differences in JP1 or STAC3 expression levels between the control and ECC‐loaded sides.

Additionally, at 3 dpi, the proportion of calpain‐1 autolysis was significantly higher on the ECC‐loaded side compared to the control side in both WT and Utrn KO mice (WT vs. WT+ECC, *p* = 0.0002; Utrn KO vs. Utrn KO+ECC, *p* = 0.0012) (Figure 6e,f). In contrast, no significant difference was observed in the proportion of calpain‐1 autolysis between the control and ECC‐loaded sides in PGC‐1α‐b Tg and PGC‐1α‐b Tg/Utrn KO mice. There was no difference in total calpain‐1 content between the groups (Figure 6g).

### 3.6 Effects of PGC‐1α‐b overexpression and eccentric contractions on the levels of proteins involved in membrane integrity

**FIGURE 7:**
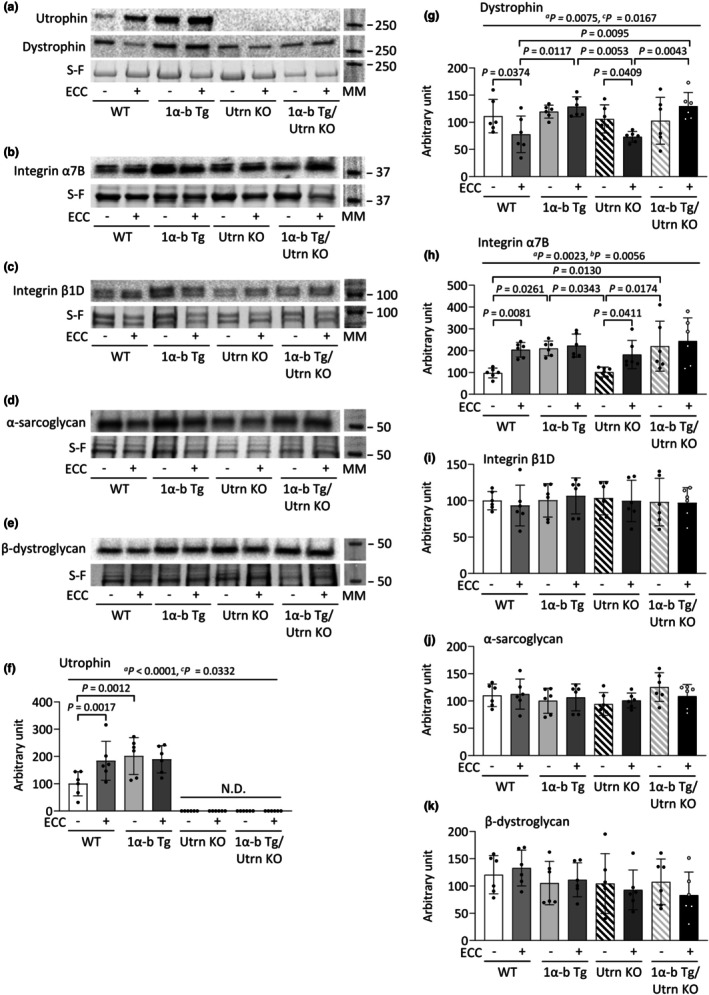
Effects of PGC‐1α‐b overexpression and eccentric contractions on the levels of costameric proteins. Representative Stain‐Free (S‐F) images and Western blots illustrating the levels of utrophin and dystrophin (a), integrin α7B (b), integrin β1D (c), α‐sarcoglycan (d), and β‐dystroglycan (e) in gastrocnemius muscles of wild‐type (WT), PGC‐1α‐b transgenic (Tg), utrophin knockout (Utrn KO), and PGC‐1α‐b Tg/Utrn KO mice at 3 days post‐injury (3 dpi) following eccentric contractions (ECCs). The levels of utrophin (f), dystrophin (g), integrin α7B (h), integrin β1D (i), α‐sarcoglycan (j), and β‐dystroglycan (k) were normalized to total protein in the corresponding S‐F images. Molecular weight markers (MM). Data show means ± SD for 6 muscles per group. Two‐way ANOVA was performed, followed by Tukey's post hoc test. Statistical significance was set at *p* < 0.05: Main effect of ^a^genotype and ^b^ECCs; ^c^interaction of genotype and ECCs; N.D. indicates not detected.

Given that PGC‐1α‐b overexpression prevented ECC‐induced sarcolemmal damage, we investigated its effects on the expression levels of proteins involved in membrane integrity. In the resting state, utrophin (Utrn) expression was significantly higher in the GST muscle of PGC‐1α‐b Tg mice compared to WT mice (*p* = 0.0012) (Figure 7a,f). At 3 dpi, ECC increased Utrn expression in WT mice (*p* = 0.0017), but not in PGC‐1α‐b Tg mice (*p* = 0.6340). At 3 dpi, both WT and Utrn KO mice showed reduced expression levels of dystrophin on the ECC‐loaded side compared to the CNT side (WT vs. WT+ECC, *p* = 0.0374; Utrn vs. Utrn KO+ECC, *p* = 0.0409) (Figure 7a,g). In contrast, this change was not observed in PGC‐1α‐b Tg or PGC‐1α‐b Tg/Utrn KO mice. Under resting conditions, PGC‐1α‐b Tg and PGC‐1α‐b Tg/Utrn KO mice exhibited increased expression levels of integrin α7B compared to WT mice (PGC‐1α‐b Tg vs. WT, *p* = 0.0261; PGC‐1α‐b Tg/Utrn KO vs. WT, *p* = 0.0130) (Figure 7b,h). Furthermore, in WT and Utrn KO mice, the expression of integrin α7B was elevated on the ECC side compared to the CNT side (WT vs. WT+ECC, *p* = 0.0081; Utrn KO vs. Utrn KO+ECC, *p* = 0.0411), whereas no such change was observed in the PGC‐1α‐b Tg+ECC and PGC‐1α‐b Tg/Utrn KO+ECC groups. Additionally, no significant differences were found among the groups in the expression levels of integrin β1D, α‐sarcoglycan, or β‐dystroglycan (Figure 7c–e,i–k).

## 4 DISCUSSION

In this study, we investigated the role of PGC‐1α‐b in resistance to muscle fiber damage using skeletal muscle‐specific PGC‐1α‐b overexpressing mice. Our results demonstrate that overexpression of PGC‐1α‐b prevents prolonged force depression following damaging ECCs in the fast‐twitch GST muscle. In WT mice, approximately 30% of muscle fibers were damaged by ECCs, with almost all of them expressing MyHC IIb. Conversely, ECC‐induced damage was largely absent in PGC‐1α‐b Tg mice, even in MyHC IIb‐positive fibers. Furthermore, these protective effects of PGC‐1α‐b were also observed in Utrn‐deficient mice. These findings indicate that PGC‐1α‐b plays a critical role in conferring resistance to ECC‐induced muscle fiber damage, independently of utrophin, which is consistent with recent reports showing that utrophin does not significantly contribute to sarcolemmal stability in dystrophin‐intact muscle (Lindsay et al., 2019). Importantly, our data also suggest a potential mechanism by which PGC‐1α‐b prevents force loss following ECCs. Specifically, PGC‐1α‐b overexpression suppressed ECC‐induced activation of calpain‐1, a Ca^2+^‐dependent protease, and prevented the degradation of key Ca^2+^ regulatory proteins such as JP1 and STAC3. These proteins are essential for E‐C coupling, and their preservation likely contributes to the maintenance of muscle force after ECCs.

In this study, biochemical analysis of MyHC isoforms using SDS‐PAGE was unable to distinguish between MyHC IIa and MyHC IIx. In contrast, immunohistochemical analysis revealed little change in the proportion of muscle fibers expressing MyHC IIb, MyHC IIa, and MyHC I in PGC‐1α‐b Tg and PGC‐1α‐b Tg/Utrn KO mice compared with WT and Utrn KO mice. Therefore, it is likely that the proportion of hybrid fibers co‐expressing MyHC IIb and IIx is increased in these mice. Our findings therefore demonstrate that overexpression of PGC‐1α‐b effectively suppresses cell membrane damage in fast‐twitch type IIb and IIb/x fibers, which are particularly susceptible to damage caused by ECCs (Choi & Widrick, 2010; Lieber & Fridén, 1988). Overall, our results underscore the importance of PGC‐1α‐b in mitigating ECC‐induced damage in vulnerable fast‐twitch muscle fibers.

Additionally, overexpression of PGC‐1α‐b increased the proportion of MyHC IIx‐positive fibers without altering the distribution of MyHC IIa‐ and MyHC I‐positive fibers, suggesting that PGC‐1α‐b overexpression does not substantially affect contraction–relaxation kinetics. Supporting this, the ratios of isometric torque at 30 Hz and 50 Hz relative to 100 Hz stimulation did not differ between the WT and PGC‐1α‐b Tg groups. On the other hand, specific isometric torque at 100 Hz was significantly lower in both the PGC‐1α‐b Tg and PGC‐1α‐b Tg/Utrn KO groups. Although the mechanism underlying this reduction in muscle strength remains unclear, similar findings have been reported in PGC‐1α1/‐a Tg mice (Yang et al., 2020). In this context, it should be considered that the reduced absolute force‐generating capacity of the PGC‐1α‐b Tg and PGC‐1α‐b Tg/Utrn KO muscles may have influenced their susceptibility to ECC‐induced damage. Because these muscles produced lower isometric and eccentric forces, the mechanical strain imposed during ECCs may have been reduced, which could have contributed, at least in part, to the attenuated damage responses observed in these groups. Therefore, the apparent resistance to ECC‐induced muscle damage should be interpreted with caution, as it may reflect altered mechanical loading conditions rather than a direct protective effect of PGC‐1α‐b per se.

Previous studies have shown that PGC‐1α1/‐a induces Utrn expression via the N‐box present in the Utrn promoter region (Angus et al., 2005). Consistent with these findings, increased Utrn expression was observed in the muscles of PGC‐1α‐b‐overexpressing mice in the present study. However, PGC‐1α‐b overexpression enhanced damage resistance in fast‐twitch fibers even in Utrn KO mice, suggesting that this effect is mediated through an Utrn‐independent pathway. Supporting this notion, Chan et al. (2014) reported that PGC‐1α1/‐a overexpression ameliorates dystrophic pathology even in mice lacking both dystrophin and Utrn.

In addition to Utrn, PGC‐1α‐b overexpression was found to increase the expression of the costameric protein integrin α7B. Boppart et al. (2008) reported that exercise promotes integrin α7B gene transcription and protects skeletal muscle. Thus, it is possible that integrin α7B may contribute to the resistance to ECC‐induced muscle damage observed with PGC‐1α‐b overexpression. However, a recent single‐fiber proteomics study revealed that costamere proteins, such as the dystrophin‐glycoprotein complex and the vinculin‐talin‐integrin system, are not necessarily less abundant in fast‐twitch compared to slow‐twitch fibers (Murgia et al., 2021), suggesting that these proteins alone are unlikely to explain the fiber type‐specific differences in susceptibility to damage.

Traditionally, excessive ECCs have been thought to cause muscle damage due to increased mechanical stress on the cell membrane (Moens et al., 1993; Petrof et al., 1993). If this were the case, membrane damage would be expected immediately after ECC loading. However, consistent with previous studies (Ashida et al., 2022; Komulainen et al., 1994; Yamada et al., 2018), histological evidence of membrane damage was scarcely observed immediately after ECC loading but became markedly apparent 3 days later. Although the mechanisms underlying this delayed onset of muscle damage remain largely unclear, the following Ca^2+^‐related hypotheses (1–3) are considered plausible: (1) an increase in intracellular Ca^2+^ concentration via stretch‐activated channels in the sarcolemma (Zhang et al., 2012) and/or Ca^2+^ release channels in the sarcoplasmic reticulum (Tabuchi et al., 2022), (2) muscle membrane lysis and the production of inflammatory cell chemotactic factors due to activation of Ca^2+^‐dependent phospholipase A_2_ (Duncan & Jackson, 1987; Howl & Publicover, 1990), and (3) amplification of secondary membrane damage caused by the release of reactive oxygen species (ROS) from infiltrating inflammatory cells (Tidball, 2011).

In this regard, PGC‐1α‐b overexpression prevented ECC‐induced activation of calpain‐1, a Ca^2+^‐dependent proteolytic enzyme, as well as the degradation of Ca^2+^ regulatory proteins such as JP1 and STAC3, both of which are known substrates of calpain‐1 (Ashida et al., 2021; Kanzaki et al., 2017; Murphy et al., 2013). These findings suggest that PGC‐1α‐b may suppress the ECC‐induced increase in intracellular Ca^2+^ concentration, potentially by modulating Ca^2+^ channels in the sarcolemma and/or sarcoplasmic reticulum. Although the precise mechanism remains unclear, these channels are likely activated by ROS, which increase in response to ECCs (Shkryl et al., 2009; Whitehead et al., 2008). Given that PGC‐1α1/‐a induces antioxidant enzymes that mitigate oxidative stress (St‐Pierre et al., 2006), it is possible that PGC‐1α‐b exerts similar effects, thereby reducing oxidative stress and preventing subsequent Ca^2+^ influx during ECCs. This antioxidant effect may also contribute to the preservation of force following ECCs, by preventing ROS‐mediated disruption of Ca^2+^ homeostasis and proteolytic damage to contractile machinery. Further investigation is needed to clarify this possibility.

On the other hand, since mitochondria can sequester Ca^2+^ via the mitochondrial Ca^2+^ uniporter, the increase in mitochondrial content resulting from PGC‐1α‐b overexpression may contribute to the suppression of intracellular Ca^2+^ elevation. However, a recent study reported that the capacity of mitochondria to sequester Ca^2+^ is limited (Lamboley et al., 2021). Furthermore, since the increase in mitochondrial Ca^2+^ content following ECCs has been shown to be associated with the opening of the mitochondrial permeability transition pore, mitochondrial Ca^2+^ uptake may contribute not to protection, but rather to tissue damage caused by mitochondrial Ca^2+^ overload (Rattray et al., 2011). Therefore, it is conceivable that the contribution of increased mitochondrial content resulting in resistance against ECCs is minimal.

## 5 LIMITATIONS

This study provides novel insights into exercise physiology; however, several limitations should be acknowledged. First, WT and PGC‐1α‐b Tg groups consisted exclusively of male mice, considering the potential for sex‐based differences in susceptibility to ECC‐induced damage (Hill et al., 2018). Although male mice were initially intended for both the Utrn KO and PGC‐1α‐b Tg/Utrn KO groups, a sufficient number of male offspring could not be obtained in the Utrn KO breeding background within the constraints of the approved experimental timeline, which necessitated the inclusion of female mice in these groups. Within the PGC‐1α‐b Tg/Utrn KO group, descriptive data are provided for both sexes for transparency; specifically, MIT (% Pre) at 3 dpi was 88.8% ± 1.1% in males (*n* = 2) and 93.8% ± 6.4% in females (*n* = 4), while the EBD‐positive area at 3 dpi was 0.09% ± 0.01% in males (*n* = 2) and 0.06% ± 0.03% in females (*n* = 4). However, given the small and unbalanced sample sizes, these observations should be interpreted with caution and do not allow statistically meaningful conclusions regarding sex‐related effects. Accordingly, the present study was not designed or sufficiently powered to assess sex differences, and potential influences of sex on ECC‐induced muscle damage cannot be excluded. Future studies using adequately powered, sex‐balanced cohorts will be required to address this issue rigorously. Second, as most assays were performed 3 days post‐injury, the study does not provide direct mechanistic insight into the early events following ECCs.

## 6 CONCLUSIONS

Our data demonstrate that PGC‐1α‐b activation contributes to resistance against ECC‐induced damage in skeletal muscle fibers. This protective effect appears to be mediated through pathways independent of Utrn expression. Fast‐twitch fibers are particularly susceptible to ECC‐induced damage, a phenomenon frequently observed in athletes (Lievens et al., 2022). Notably, previous studies have shown that PGC‐1α‐b expression increases with exercise in an intensity‐dependent manner (Tadaishi, Miura, Kai, Kawasaki, et al., 2011). These findings suggest that targeted exercise strategies aimed at enhancing PGC‐1α‐b expression may modulate skeletal muscle responses to ECC–induced stress.

## AUTHOR CONTRIBUTIONS

T.Y. conceived and designed research; A.N., N.T., N.Y., A.N., K.O., K.H., Y.A., and T.Y. performed experiments; A.N., N.T., N.Y., A.N., K.O., K.H., Y.A., and T.Y. analyzed data; A.N., N.T., N.Y., A.N., K.O., K.H., Y.A., and T.Y. interpreted results of experiments; A.N. and T.Y. prepared figures; A.N. and T.Y. drafted manuscript; T.Y. edited and revised manuscript; all authors approved the final version of the manuscript.

## FUNDING INFORMATION

This work was supported by grants from the Japan Society for the Promotion of Science (JP25K02981; to T.Y.).

## CONFLICT OF INTEREST STATEMENT

No conflicts of interest, financial or otherwise, are declared by the author(s).

## Supporting information


Appendix S1.

## Data Availability

Data will be made available upon reasonable request.
