# Supplementary material for: Skeletal muscle‐specific PGC‐1α‐b overexpression prevents eccentric contraction‐induced muscle injury through an utrophin‐independent pathway in mice
Source: Physiol Rep. 2026 Jan 22;14(2):e70743. doi: 10.14814/phy2.70743 (PMC12828171; doi:10.14814/phy2.70743)

Supplementary Figure 1. Original uncropped blot image used for Figure 1E and G

Fig. 1E

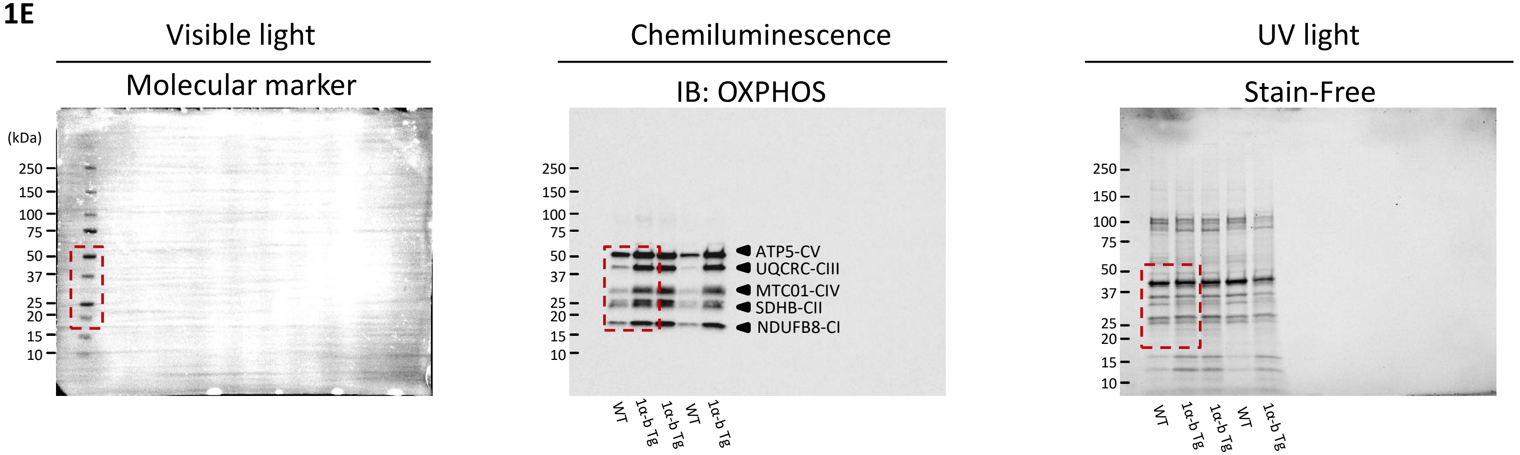

Fig. 1G

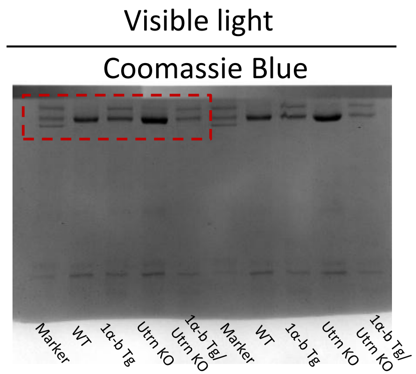

Note: This method is an SDS-PAGE technique developed for the separation of MyHC isoforms and does not require molecular weight markers.

Supplementary Figure 1. Original uncropped blot image used for Figure 6A & C

Fig. 6A

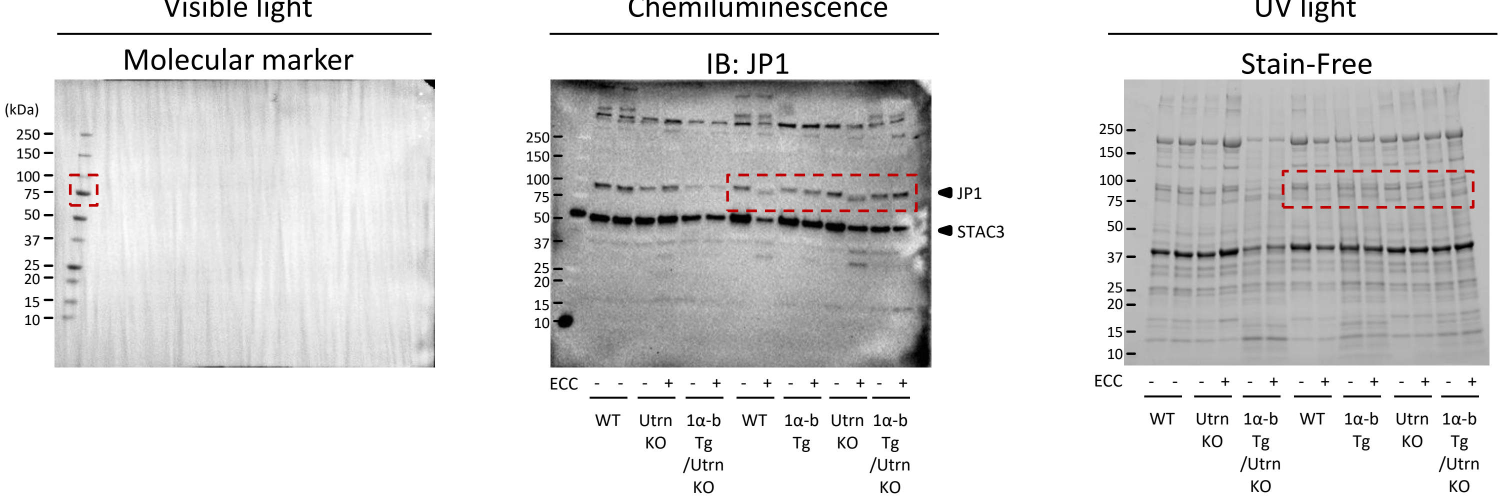

Fig. 6C

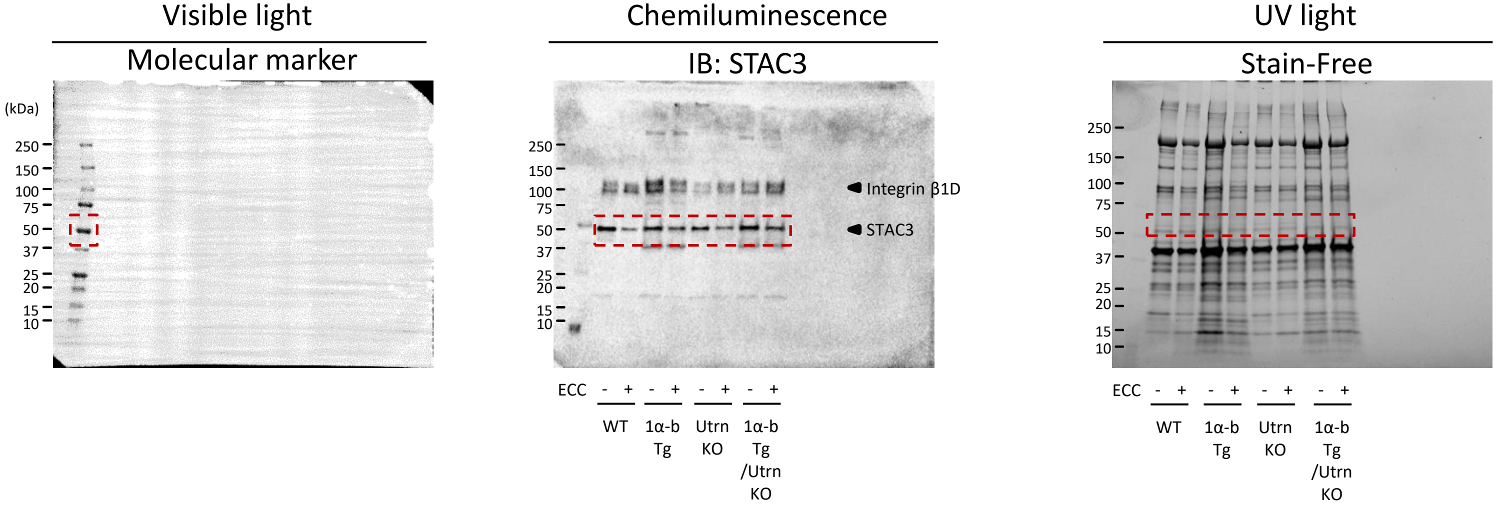

Supplementary Figure 1. Original uncropped blot image used for Figure 6E

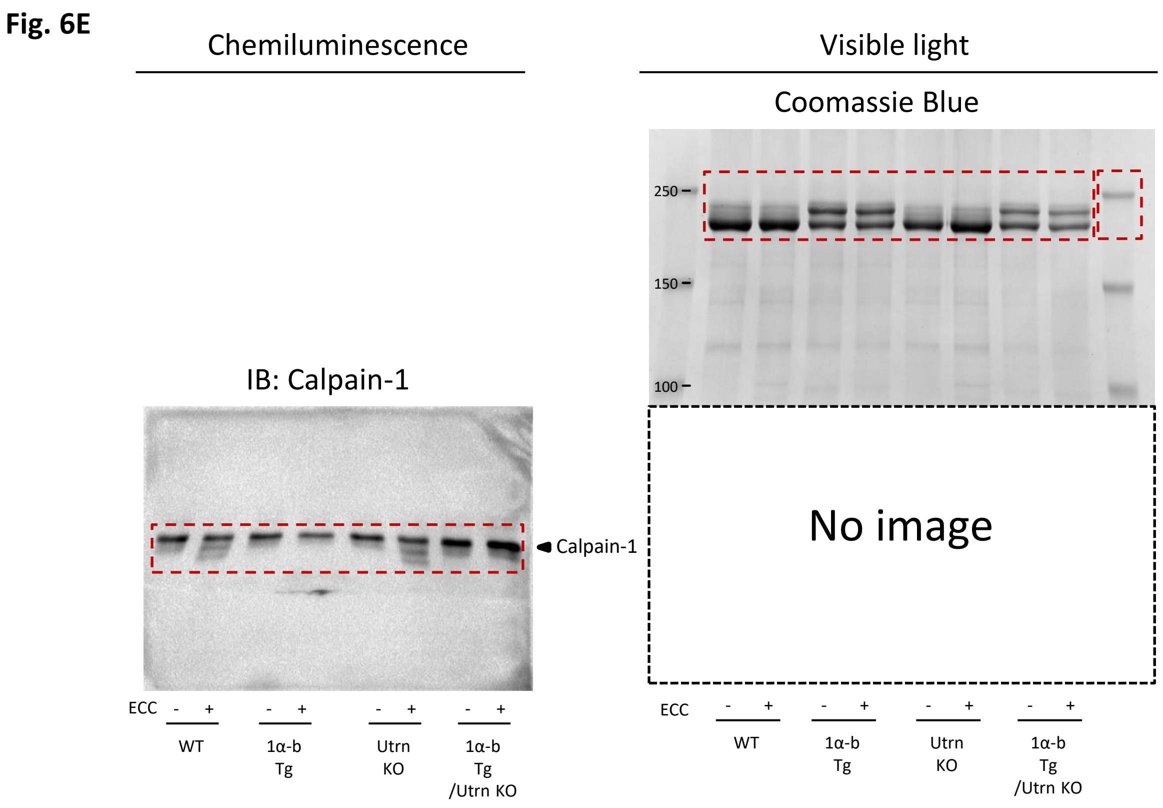

Note: The samples were separated using 7% SDS-PAGE and divided into upper and lower gel sections. The upper section (>100 kDa) was stained with Coomassie Brilliant Blue to visualize MyHC and used as a loading control. In contrast, the lower section (<100 kDa) was transferred to a membrane and subjected to Western blotting using an anti-calpain-1 antibody. Since the lower gel was not stained prior to membrane transfer, no image is available for that section.

Supplementary Figure 1. Original uncropped blot image used for Figure 7A

Fig. 7A

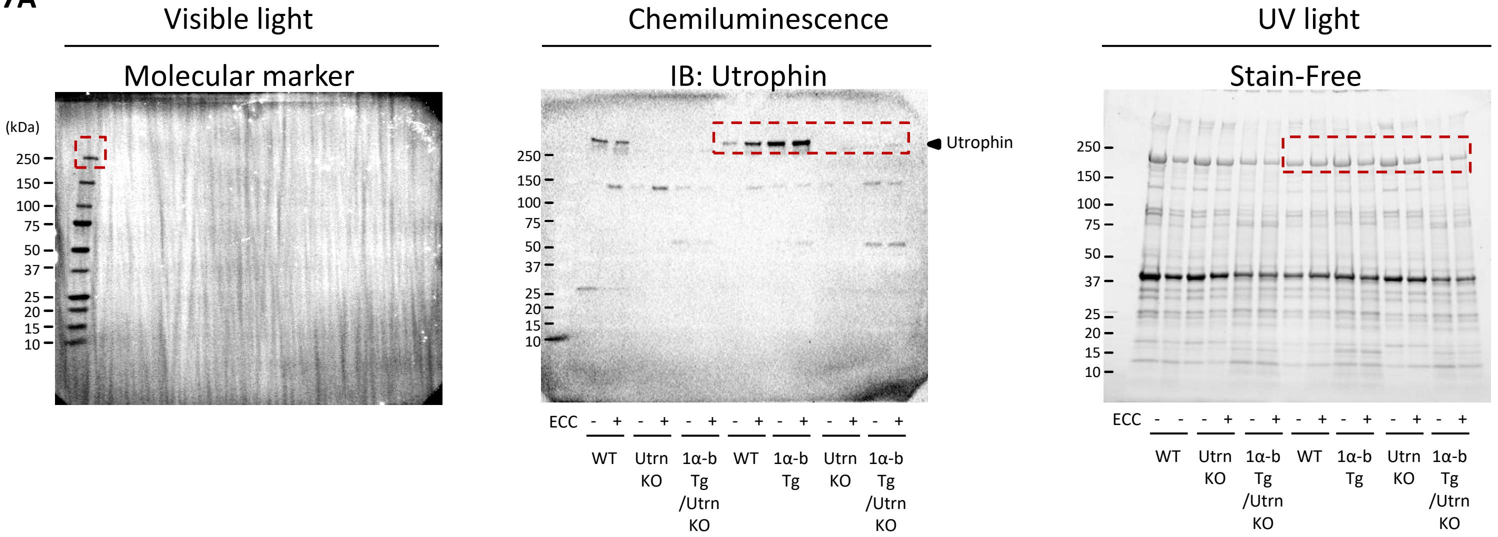

Fig. 7A

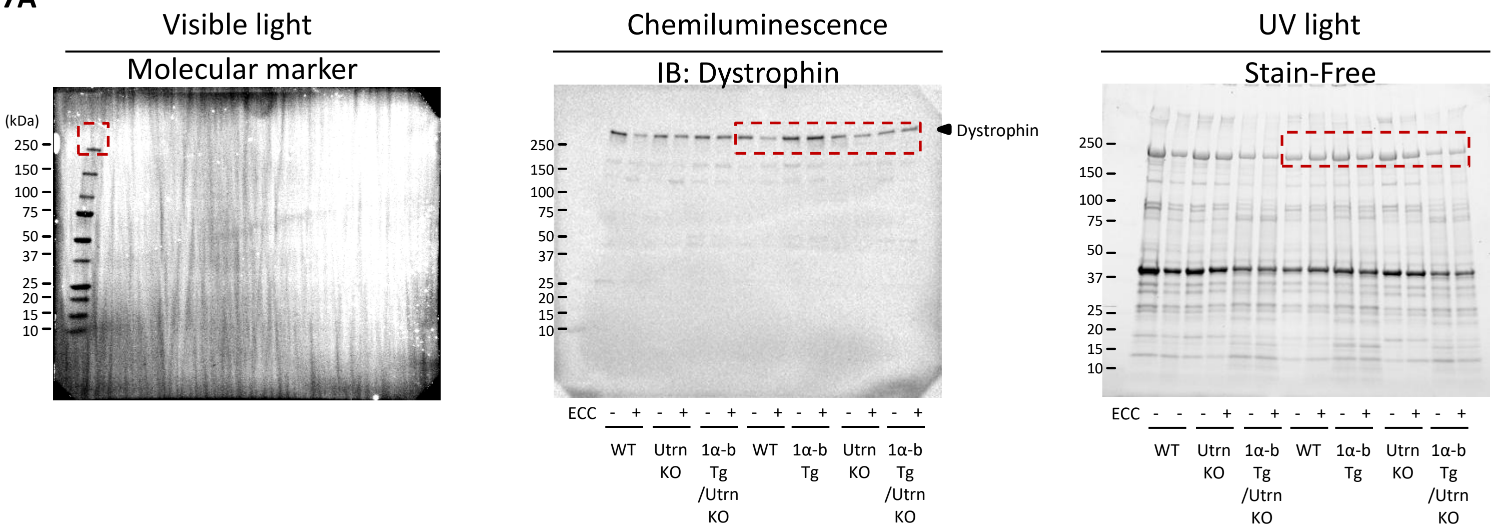

Supplementary Figure 1. Original uncropped blot image used for Figure 7B & C

Fig. 7B

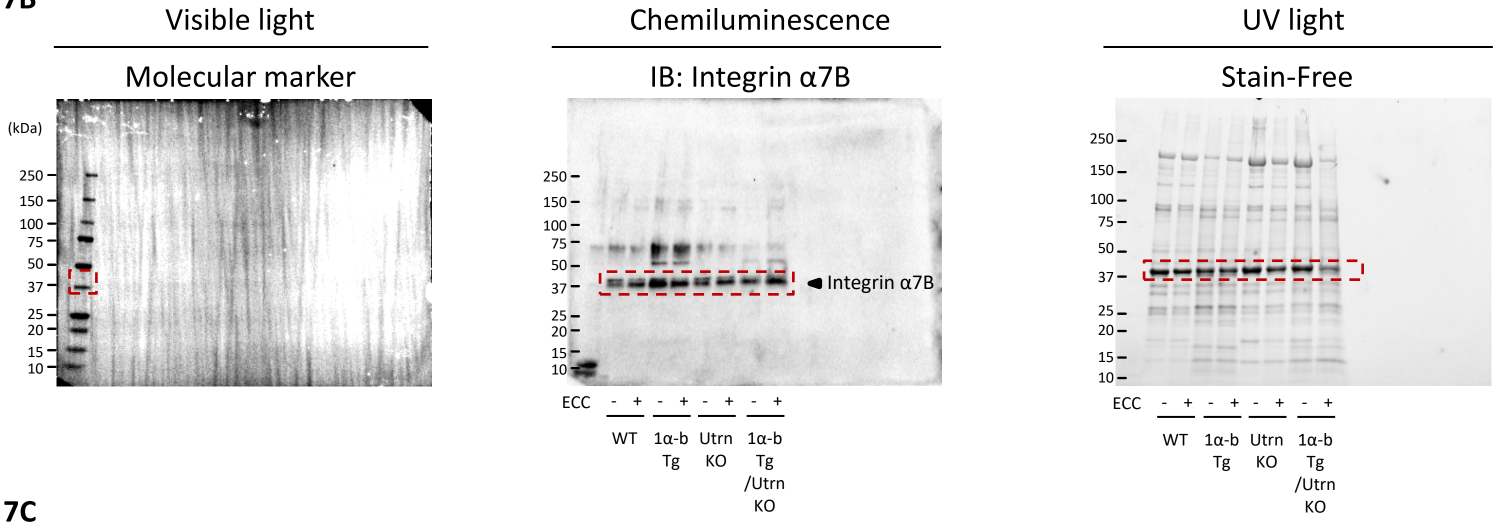

Fig. 7C

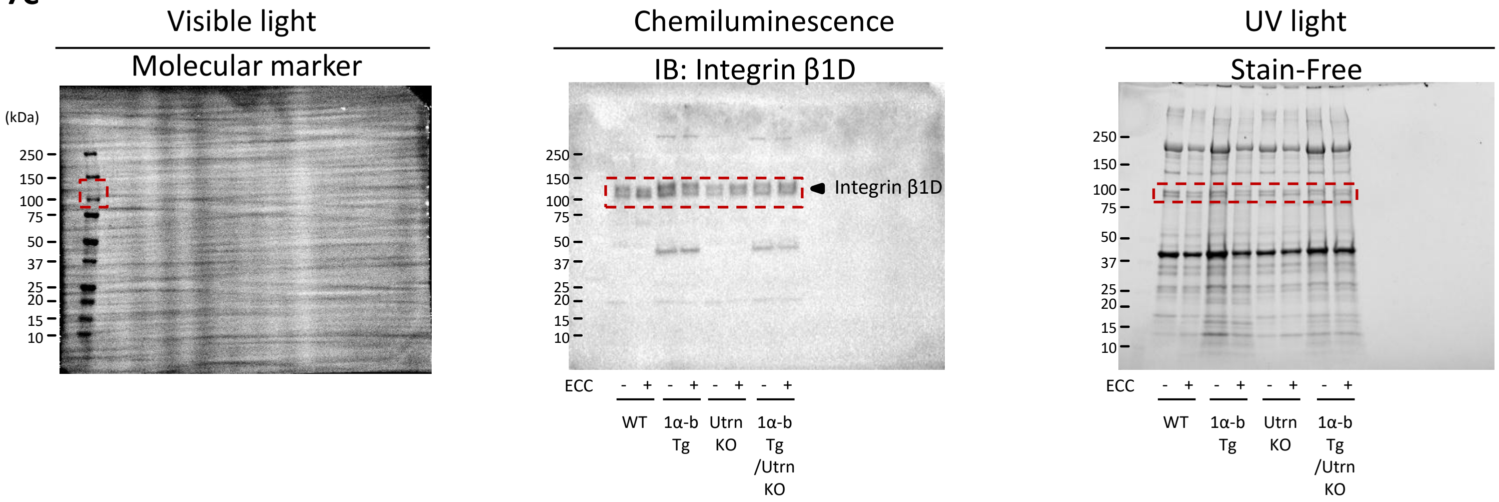

Supplementary Figure 1. Original uncropped blot image used for Figure 7D & E

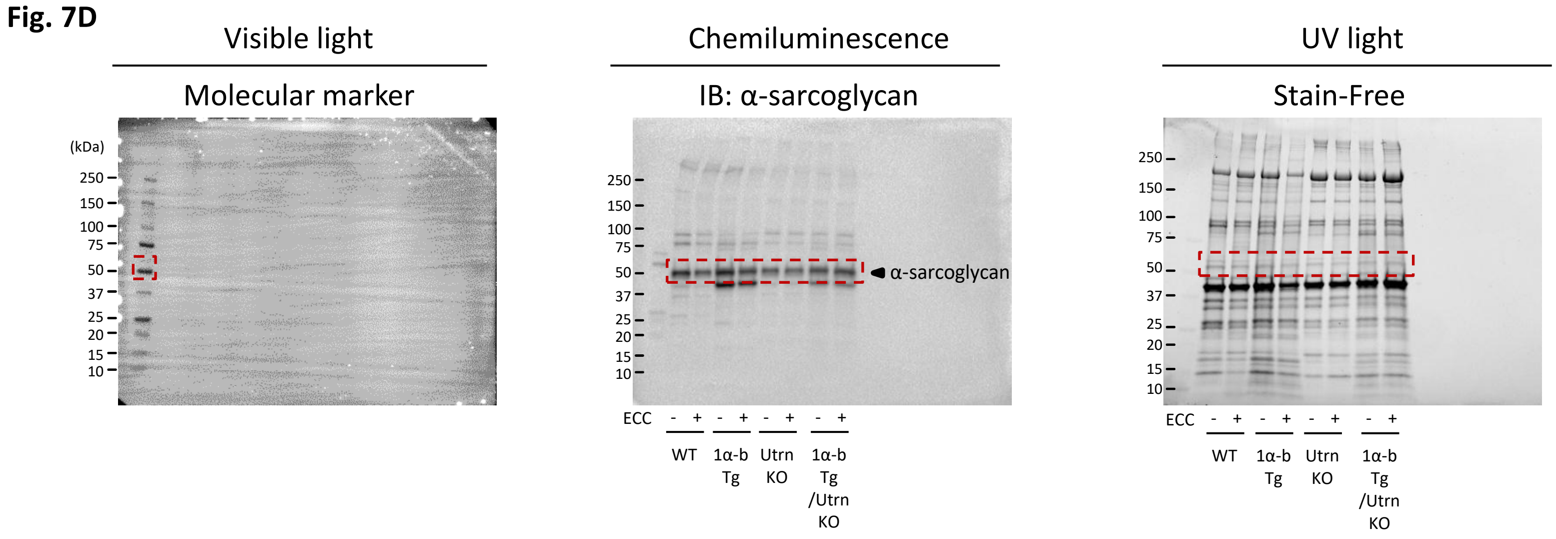

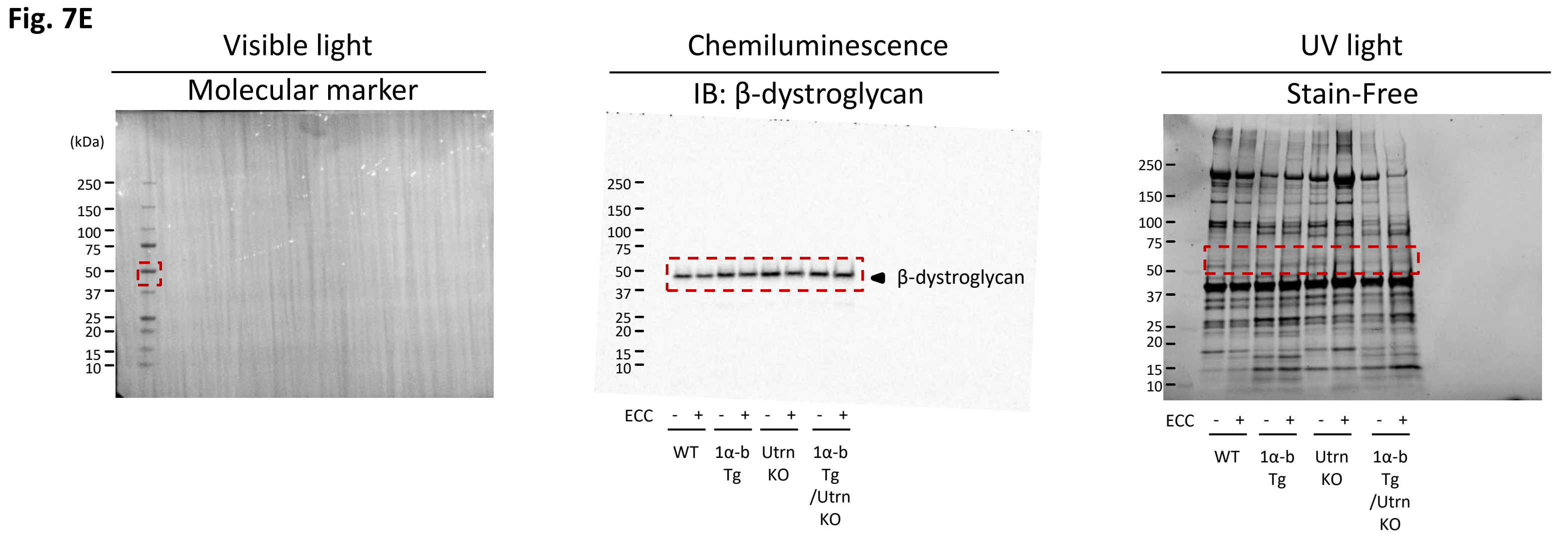

Supplement: Supplementary file 1 — Appendix S1. [file PHY2-14-e70743-s001.pdf]
